# P66SHC and Ageing: ROS and TOR?

**DOI:** 10.18632/aging.100182

**Published:** 2010-07-28

**Authors:** Giovambattista Pani

**Affiliations:** Institute of General Pathology, Universita' Cattolica Medical School, Rome Italy

**Keywords:** p66shc, mTOR/S6K, ageing, calorie restriction, nutrient-sensing, diabetes

## Abstract

Both Reactive Oxygen Species (ROS) and hyperactivation of the nutrient-sensing mTOR/S6 kinase cascade have been linked to aging and age-related diseases  as well as to the anti-aging effect of calorie restriction.  Recent findings that the pro-aging and pro-oxidant molecule p66shc contributes to S6K activation by nutrients and promotes insulin resistance and diabetes in mice may provide an answer to the "ROS or TOR?" dilemma.

In late 90's, the idea that manipulation of one single gene could significantly extend longevity of a complex model organism was certainly not an heretic one [1]. Successful attempts had already been made in worms and flies, not to speak about budding yeast, whose unicellular simplicity makes it somehow close to a cell culture system. It was already clear, in particular, that hypomorphic mutations in the insulin/Igf1 (IIS) pathway could enhance lifespan in Drosophila or C. Elegans, in a fashion that could mimic the effect of nutrient restriction, for the longest time the most reliable model for laboratory research on longevity.  The role of insulin/Igf signaling in mediating body response to nutrients, and the fact that IIS is reduced by calorie restriction protocols, provided full rationale to these observations, that brought to the notion that aging may represent, rather than the uncontrolled catastrophe of the body, the product of a genetically coded programme.  Yet, report by Migliaccio and colleagues,  that mice lacking the 66kD isoform of the Shc (Src Homology and Collagen) protein family lived 30% longer than p66-proficient littermates,  caught  the scientific community by surprise, especially because p66KO mice, not only were long lived, but appeared, unlike other murine models of longevity such as GH deficient dwarf mice, phenotypically normal, fertile and healthy [2].

Shc proteins were known as adapter molecules, i.e. signaling components deputed to the assembly of macromolecular complexes downstream of activated growth factor receptors (RTKs). A role for SHCs in insulin signaling, in particular, had also been reported [3]. Thus, one could have easily welcomed the p66KO mouse as the first (or one of the first) mammalian example(s) of extended longevity by genetic attenuation of insulin/Igf signaling. Another one, the Igf-1 receptor (IGF-1R)  knock-out mouse, was going to come shortly after [4].

Instead, the linkage between p66 and longevity took an unexpected direction, becoming one of the strongest arguments in support of the Harman's "free radical theory of aging" [5]: in fact, p66- deficient mice and cells were found to present remarkably reduced levels of ROS and increased resistance to oxidative stress.

Attenuation of insulin signaling leads per se to reduced oxidative burden, by Daf-16/FoxO dependent up-regulation of antioxidant defenses [6]; thus, the oxidant-resistant phenotype of p66KO mice could still fit in the genetic model of longevity centered on the insulin/Igf signaling cascade. Instead, second surprise, solid biochemical studies revealed for p66shc a function completely distinct from that of the other SHC proteins: it was found that, in response to a number to pro-oxidant and apoptogenic stimuli, p66shc translocates to mitochondria, where it directly generates reactive oxygen species, by transferring electrons from cytochrome c to oxygen [7]. This finding tied p66 and its effect on longevity to ROS and mitochondria, in perfect agreement with Harman's theories; accordingly, studies performed on p66KO mice involved p66 in a number of typical age-related diseases, including vascular diabetic complication and atherosclerosis, already suspected to be caused by excess oxidative stress [8].  Interestingly, in keeping with initial predictions, insulin signaling was indeed found to be defective in p66-deficient cells and mice, but that was again related to the molecule's capacity to generate ROS, that facilitate tyrosine kinase signaling by transient and reversible inhibition of tyrosine phosphatases [9].

Accumulation of cellular and tissue oxidative damage, however, may nor represent the only, or even the most important, mechanism underlying body senescence and limitation of lifespan. Mounting evidence indicate that  effects of calorie restriction on longevity involve a number of nutrient-sensing molecular networks that regulate, beside ROS generation and scavenging, also DNA repair, inflammation, cell proliferation and body growth (i.e. accumulation of  biomass) [10]. One of these evolutionarily conserved networks involves the sirtuin family of NAD+ dependent histone deacetylases (sirtuin 1 through 7 in mammals) that regulate chromatin remodelling and gene transcription in response to cellular energy status [11]. Another major nutrient-sensing pathway is centered on the TOR (Target of Rapamycin) kinase and its downstream cascade. In mammalian cells, m(ammalian)TOR regulates ribosomal protein synthesis, cell growth, cell cycle progression, autophagy and mitochondrial function in response to the availability of aminoacids and the intracellular levels of ATP. Additionally, mTOR is activated by growth factor receptors. Including, of course, the insulin receptor [12].

Several lines of evidence indicate that nutrient and insulin-dependent regulation of TOR and its downstream cascade may play a central role in aging and in the nutritional control of lifespan. In yeast, flies and worms, hypomorphic mutations in this cascade extend longevity [10]. Even more interestingly, the mTOR inhibitor Rapamycin  extends lifespan in mice and prevents age-related diseases [13], and so does genetic deletion of the ribosomal S6 kinase (S6K), a major downstream effector of mTOR [14]. Thus, inactivation of the mTOR pathway mimics the beneficial effect of calorie restriction in rodents, clearly indicating that mTOR-dependent signaling contributes to longevity determination by nutrients in mammals. Again, inhibition of TOR may lead to increased antioxidant defenses, as observed in yeast and flies [15], but could also promote autophagy and reduce  intracellular accumulation of pathologic proteins, that eventually leads to  Endoplasmic Reticulum (ER) stress and tissue aging [16]. Notably, accumulation of misfolded proteins underlies typical senescence-associated pathologies like Alzheimer's and vascular  amyloidosis, while ER stress contributes to insulin resistance and Metabolic Syndrome, another age-dependent disease [17].

Is there a relationship between p66-dependent aging and the regulation of longevity by nutrients, through the  mTOR/S6K cascade? Or, in other words, does the mTOR/S6K cascade contribute to p66 effects on mouse lifespan? Recent work performed in our laboratory tried to address this seemingly relevant  question [18].

We were initially interested in determining whether p66shc may have a role in insulin resistance, the signaling dysfunction underlying glucose intolerance and type 2 diabetes associated with overnutrition and overweight. The question was legitimated by increasing evidence of a role for reactive oxygen species in insulin desensitization [19], and by our previous observation of reduced liver steatosis, a major inducer of insulin-resistance, in p66KO mice [20]. We indeed found that  obese (LepOb, leptin deficient) mice devoid of  p66, although gaining nearly as much weight as their p66-proficient littermates, remained remarkably responsive to insulin and were significantly protected from diabetes. Importantly, this finding correlated with reduced levels of phosphorylation of S6K in the adipose tissue; additionally, isolated adipocytes from p66KO obese mice displayed reduced S6K activity and preserved insulin responsiveness compared to p66 WT cells, and p66KO preadipocytes were resistant to the insulin-desensitizing effect of excess fatty acids *in vitro*.

These findings fitted with the current model whereby excess nutrient (glucose and Free Fatty Acids) and chronic hyperinsulinemia downregulate insulin response in target tissues by hyperactivating S6K, that in turn leads to serine phosphorylation and proteasomal degradation of the major insulin transducer IRS-1 [21]. p66 would participate in this circuitry by somehow stimulating S6K. Accordingly, we showed that overexpression of p66shc in 3T3L1 adipocytes leads to hyperactivation of S6K and to hyperphosphorylation of IRS on serine residues. Further molecular dissection of these biochemical events also revealed that p66shc forms a complex with S6K 1 and IRS-1, thus facilitating the signal-inibitory interaction between the two molecules. To our surprise, these effects of p66 were largely independent from changes in the intracellular redox state, or from the redox properties of p66 itself, but seemingly explainable by the "traditional" function of p66shc as an adapter protein. We concluded that p66shc, at least in adipocytes, promoted insulin and nutrient signaling to S6K, and, consequently, the feed-back inhibitory action of S6K on IRS-1, leading to diabetes in overfed animals.

While these findings have obvious relevance for the understanding of signal deregulation connecting obesity and overnutrition to diabetes, our observation may add a novel  perspective to the linkage between p66shc and lifespan determination.

In fact, ablation of p66, by leading to reduced responsiveness  of S6K to nutrients, creates a Rapamycin-like (although presumably milder) signaling block that conceivably promotes animal longevity,  at least by preventing one major age-related disease, type 2 diabetes. In simpler words, p66 ablation could mimc calorie restricttion. Notably type 2 diabetes recapitulates and accelerates many pathologic changes (in vasculature, kidneys, eyes, peripheral nerves) that are typical of senescence. These changes hit tissues that are largely insulin-independent for their energy metabolism, but that are exposed to elevated amount of insulin and glucose imposed by whole body insulin resistance. Interestingly, obese mice lacking p66 live significantly longer than their p66WT controls (although less than lean, WT mice) [17]. On the other hand, laboratory animals fed *ad libitum* frequently develop overweight and glucose intolerance with age, indicating that effects of p66shc observed in the context of genetic obesity and diabetes  may also be relevant to the aging process of non overtly obese mice.

**Figure 1.**
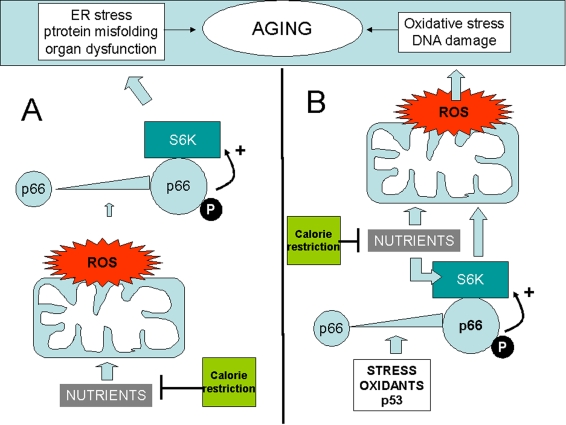
Two distinct models whereby p66shc may integrate ROS and the TOR/S6K cascade in the aging process. (**A**) ROS upregulate  p66shc and activate S6K through p66. Oxidant species could be generated by mitochondria in response to nutirents, thus creating an alternative route for nutrient sensing by S6K. (**B**) S6K, activated by p66, increases ROS formation in mitochondria. In this case p66 could be in turn activated by  cellular stress, by p53, or  by environmental oxidants.  In both examples p66 effects on aging are inhibited by calorie restriction  (green box) that reduces nutrient supply. Activation" of p66shc is depicted as a result of increased expression (larger icon) and serine phophorylation (letter "P").  Both changes have in fact been reported in response to diverse stresses in mammalian cells.

Apart from prevention of glucose dysmetabolism, all the S6K-related mechanisms for lifespan extension may operate, in view of our findings, in p66KO mice. For instance, reduced protein translation may attenuate ER stress in critical tissues and reduce progression and severity of  age-related diseases due to accumulation of misfolded proteins. While this possibility deserves to be tested in appropriate model systems (such as mice prone to  Alzheimer's disease crossed to p66KO mice), we have preliminary evidence that overexpression of p66shc in preadipocites and kidney cells increases ER stress in parallel with hyperactivation of S6K.

Along similar lines, increased autophagy, due to S6K attenuation, may contribute to the long-lived phenotype of p66 deficient animals, another possibility to be verified.

Finally, prevention of cancer contributes to lifespan extension by calorie restriction and S6K blockade. This may be true also in p66KO mice. Interestingly, in spite of p66shc operating in the p53-initiated apoptotic pathway [22], no increase in tumor incidence has been described in this mouse strain. Based on our prediction such incidence may even be lower than in wild type animals, due, at least in part, to reduced mTOR/S6K signaling in cancer cells. This is again a testable hypothesis.

Can these views be reconciled with current, "ROS-centric" model for lifespan limitation by p66 [23]? In principle, ROS can operate both upstream and  downstream of  the TOR cascade. In one scenario, p66 action on S6K may lead to increased mitochondrial metabolism and as a consequence to a rise of mitochondrial ROS [24], as observed in cells were p66shc is overexpressed [2]. In simple terms, mTOR/S6K may mediate, at least in part, the pro-oxidant action of p66 (Figure 1B).

More intriguingly, ROS may act upstream of the p66/S6K module, since p66shc not only generates ROS, but is also stimulated by oxidants [2]. For instance, in fibroblasts exposed to oxidative stress, PI3K/AkT activation by ROS is mediated, at least to some extent, by p66shc [25]; AkT can, in turn, activate mTOR.  ROS are also generated in mitochondria in response to energy substrates; these species may increase the phospho-rylation/expression level of p66, thereby promoting  its (redox-independent) stimulatory action on  S6K.  This would represent  an intriguing alterantive route for nutrients to signal, *via* mitochondria, ROS and p66shc, to the mTOR/S6K cascade (Figure 1A).   Of note, phosphorylation of p66, a modification that correlates  with its biological activity, was found to be increased in pre-adipocytes exposed to hyperglycemia or excess FFA, as if p66 were actually behaving  as a  sensor of nutrient abundance in these cellular contexts [17].

In all the above scenarios, p66, S6K and ROS lie on the same nutrient sensitive pathway, mechanistically linked  to aging and potentially targetable by calorie restriction (Figure 1 A and B).

In conclusion, the observation that p66shc contributes to S6K activation in response to glucose, amino acids and insulin, supports the concept that aging and age-related diseases  are driven by TOR (not by ROS) and p66sch accelerates aging by activating TOR [26]; revealing the  existence of a  novel nutrient-regulated pathway to senescence,  in which p66shc works as an adaptor (what else?) between ROS *and* TOR.
